# SDF‐1/CXCR4 signalling is involved in blood vessel growth and remodelling by intussusception

**DOI:** 10.1111/jcmm.14269

**Published:** 2019-04-04

**Authors:** Ivanka Dimova, Swapna Karthik, Andrew Makanya, Ruslan Hlushchuk, David Semela, Vladislav Volarevic, Valentin Djonov

**Affiliations:** ^1^ Institute of Anatomy, University of Bern Bern Switzerland; ^2^ Center of Molecular Medicine Medical University Sofia Sofia Bulgaria; ^3^ Department of Veterinary Anatomy and Physiology University of Nairobi Nairobi Kenya; ^4^ Liver Biology Laboratory, Medical Research Center Cantonal Hospital St. Gallen St. Gallen Switzerland; ^5^ Center of Molecular Medicine and Stem Cell Research, Faculty of Medical Sciences University of Kragujevac Kragujevac Serbia

**Keywords:** bone marrow‐derived mononuclear cells, intussusceptive angiogenesis, SDF‐1/CXCR4 signalling, vessel remodelling

## Abstract

The precise mechanisms of SDF‐1 (CXCL12) in angiogenesis are not fully elucidated. Recently, we showed that Notch inhibition induces extensive intussusceptive angiogenesis by recruitment of mononuclear cells and it was associated with increased levels of SDF‐1 and CXCR4. In the current study, we demonstrated SDF‐1 expression in liver sinusoidal vessels of Notch1 knockout mice with regenerative hyperplasia by means of intussusception, but we did not detect any SDF‐1 expression in wild‐type mice with normal liver vessel structure. In addition, pharmacological inhibition of SDF‐1/CXCR4 signalling by AMD3100 perturbs intussusceptive vascular growth and abolishes mononuclear cell recruitment in the chicken area vasculosa. In contrast, treatment with recombinant SDF‐1 protein increased microvascular density by 34% through augmentation of pillar number compared to controls. The number of extravasating mononuclear cells was four times higher after SDF‐1 application and two times less after blocking this pathway. Bone marrow‐derived mononuclear cells (BMDC) were recruited to vessels in response to elevated expression of SDF‐1 in endothelial cells. They participated in formation and stabilization of pillars. The current study is the first report to implicate SDF‐1/CXCR4 signalling in intussusceptive angiogenesis and further highlights the stabilizing role of BMDC in the formation of pillars during vascular remodelling.

## 1 INTRODUCTION

Angiogenesis is essential for normal embryonic development, and plays a key role in pathological conditions related to tumour growth and ischaemic cardiovascular diseases. This is a complex process involving essential signalling pathways such as VEGF, basic fibroblast growth factor (bFGF) and Notch in vasculature. Previously published results suggested that myeloid progenitor cells play important role in angiogenesis.^1^

We have largely expanded our knowledge about the role of bone marrow‐derived cells (BMDC) in stimulating angiogenesis after their discovery in 1997^2^ and now their capability to promote vessel formation is intensively investigated. The domain comes to be multifaceted and contradictory data were sometimes arising. The discovery that mononuclear cells can home to sites of hypoxia and enhance neo‐angiogenesis has faced the possibility of using isolated hematopoietic stem cells or endothelial progenitor cells (EPC) for therapeutic vasculogenesis.^3^ However, infusion of EPC did not improve neovascularization^4^, ^5^ suggesting that a not‐yet‐defined functional characteristic (eg, chemokine or integrin receptors mediating homing) is essential for EPC‐mediated vascular augmentation after ischaemia.^6^ During endothelial repair after vascular injury and during tumour angiogenesis, BMDC do not seem to be involved in re‐endothelialization, stressing their supportive role over trans‐differentiation.^7^, ^8^ For therapeutic application, local enhancement of monocyte recruitment might be better suited than systemic infusion of monocytic cells, which only leads to a relatively minor increase in the number of circulating monocytes. An open question is what drives BMDC recruitment to the sites of angiogenesis.

Ischaemia is believed to up‐regulate VEGF or SDF‐1 (CXCL12),^9^ the latter in turn is released to the circulation and induces mobilization of progenitor cells from the bone marrow via a MMP‐9—dependent mechanism.^10^, ^11^ Indeed, SDF‐1 has been proven to stimulate recruitment of progenitor cells to the ischaemic tissue.^12^ SDF‐1 protein levels were increased during the first days after induction of myocardial infarction.^13^ Moreover, overexpression of SDF‐1 augmented stem cell homing and incorporation into ischaemic tissues.^12^, ^13^ Interestingly, hematopoietic stem cells were shown to be exquisitely sensitive to SDF‐1 and did not react to G‐CSF or other chemokines (eg, IL‐8 and RANTES).^14^

SDF‐1/CXCR4 axis is crucial in the homing mechanisms of hematopoietic cells and metastasis of solid tumours. In the past few years, numerous studies have focused on unravelling the role of this signalling in angiogenesis and prove its angiogenic activity in organ repair and tumour development. However, the precise mechanisms by which SDF‐1 exerts its pro‐angiogenic effects are not fully elucidated. Since it is supposed to be an angiogenic growth factor, it is a good candidate for pro‐ and anti‐angiogenic therapy. It was reported that transient disruption of the SDF‐1/CXCR4 axis using CXCR4 blocking antibody blocked the recruitment of bone marrow‐derived cells into the angiogenic sites of tumour tissue, and resulted in an inhibition of accelerated tumour growth.^15^

Recently we have shown that inhibition of Notch signalling induces extensive intussusceptive angiogenesis by recruitment of mononuclear cells.^16^ Notably, it was associated with increased levels of SDF‐1 and CXCR4 chemotaxis factors. Intussusceptive angiogenesis is a process linked to both blood vessel replication and remodelling in development.^17^, ^18^ It is well documented as a mechanism of vascular adaptation in response to different environmental stimuli such as chronic systemic hypoxia^19^ and prolonged inflammation.^20^ It is also a mechanism of compensatory vascular growth. For example, capillary repair during kidney recovery in Thy1.1 nephritis proceeds through intussusceptive angiogenesis.^21^ Similarly, the switch from sprouting to intussusceptive angiogenesis, found to occur in tumours after irradiation therapy, allows the vasculature to maintain its functional properties.^22^, ^23^ Potential candidates for molecular targeting of this angioadaptive mechanism are yet to be elucidated in order to improve the currently poor efficacy of contemporary anti‐angiogenic therapies. Of major significance is the involvement of intussusceptive angiogenesis in pathological conditions such as liver nodular hyperplasia,^24^ in the vasculature of experimental and clinical tumours,^25^, ^26^ in liver metastasis,^29^ in metastatic tumours of the brain^30^ and in breast cancer progression^31^ among others.

Despite this variety of roles, attributed to intussusceptive angiogenic, most of the current research is focused on sprouting angiogenesis because the latter mechanism has been known since many decades ago and additionally, there are many experimental models related to sprouting angiogenesis. Here, we provide evidence that intussusceptive angiogenesis is regulated by SDF‐1/CXCR4 signalling and suggest some intussusceptive angiogenic roles for CXCR4 and Tie‐2 positive bone marrow‐derived mononuclear cells (BMDC).

## 2 METHODS

### 2.1 Animals

Fertilized white leghorn eggs were obtained from commercial breeders (Fribourg, Switzerland). The eggs were incubated in‐shell for 3 days at 37°C in humidified (65%) atmosphere, containing 1%‐2% CO_2_. The eggs were opened on day 3 and further incubated at the same conditions using shell‐free method^32^ in petri dishes (Corning Incorporated, Corning, NY). The samples were divided into a control and experimental groups. There were at least six chicken embryos investigated in each group.

MxCre Notch1lox/lox mice on a C57Bl/6 background carrying the Cre‐recombinase under the murine Mx1 promoter (described in 24). To induce recombination, 300 µg of polyiosinic‐polycytidylic acid (pIpC) (InvivoGen, San Diego, CA) was injected intraperitoneally in 4‐week‐old mice at days 0, 3 and 6, resulting in efficient deletion of Notch1 in the liver already after 1 day. Notch1 deletion was consistent in liver sinusoidal endothelial cells (LSECs) and hepatocytes during the whole observation period.

### 2.2 Reagents

Inhibition of Notch signalling was achieved as already described (Dimova et  al) by using γ‐secretase inhibitor 1 (GSI‐1) (Calbiochem, San Diego, CA, USA). To block SDF‐1/CXCR4 signalling, an antagonist of CXCR4—a symmetrical bicyclam compound AMD3100 (Calbiochem), which inhibits SDF‐1 activation of the receptor was used, in a dose of 40‐80 mg/kg. AMD3100 application was done subsequent to GSI treatment. To stimulate SDF‐1/CXCR4 signalling, recombinant SDF‐1 alpha protein (PromoKine, Heidelberg, Germany) was used at a dosage of 10 mg/kg, with two times of application during 24 hour‐period of observation in chicken area vasculosa (CAV).

### 2.3 Morphometry

Evaluation of vascular parameters was accomplished with Tem Imaging Platform software (iTEM). Electronic images were acquired from normally developing CAV in order to obtain baseline data and a normal growth curve. From the experimental groups, images were taken from treated samples at time point 24 hours. The visualization of microvasculature was done by injection of 50 µL of 10%‐fluorescein isothiocyanate‐Dextran 2000 kD (FD‐2000S; Sigma‐Aldrich, Munich, Germany) in 0.9% NaCl solution, prior to inspection in an epifluorescence microscope (Polyvar‐Reichert, Glattbrugg, Switzerland) equipped with a Canon 5D Mark II camera for both video recording and acquisition of still images. The still images and video sequences of at least four fields of view were obtained per application site for further quantitative evaluation. Microvascular density and pillar numbers were evaluated using analySIS Software 5.0 (Soft Imaging System, Muenster, Germany).

### 2.4 Transmission electron microscopy

The samples obtained from the area vasculosa and PBS controls harvested at time point 24 hours were immersion‐fixed in 2.5% glutaraldehyde solution buffered with 0.03 mol/L potassium phosphate (pH 7.4, 370 mOsm). The livers of Notch 1 KO and wild‐type (WT) mice were perfused with 2.5% glutaraldehyde in 0.1 mol/L cacodylate buffer [pH 7.4, 350 mOsm]). Then the livers were excised, cut into small pieces, and immersed in the same fixative.

All samples were then postfixed in 1% OsO4 (buffered with 0.1 mol/L sodium cacodylate—pH 7.4, 370 mOsm), dehydrated in ascending concentrations of ethanol, and embedded in epoxy resin. For light microscopy, 1µm‐thick sections were prepared using glass knives and stained with toluidine blue. For transmission electron microscopy, 80‐90 nm‐thick sections were prepared and mounted on copper grids coated with Formvar (polyvinyl formal; Fluka, Buchs, Switzerland). They were stained with lead citrate and counterstained with uranyl acetate prior to viewing in a Philips EM‐400 electron microscope.

### 2.5 Vascular casting

Vascular casts were prepared as previously described.^33^ Briefly, CAV vasculature and the vasculature of the murine livers were perfused with a freshly prepared solution of Mercox^®^ (Vilene Company, Tokyo, Japan) containing 0.1 mL of accelerator per 5 mL of resin. One hour after perfusion, the specimes were transferred to 7.5% potassium hydroxide for digestion of tissue, which was effected over a course of 2‐3 weeks. After washing, the casts were dehydrated in ethanol and dried in a vacuum desiccator. The samples were then sputtered with gold to a thickness of 10 nm and examined in a Philips XL‐30 SFEG scanning electron microscope.

### 2.6 Mononuclear cell counting

Semithin serial sections were obtained and images captured at magnification 40x using a light microscope (Leica, Leitz DM), equipped with Leica DFC480 camera. At least 10 images were taken per sample for further quantitative evaluation and at least 10 samples for each application were evaluated. The total number of adherent/extravasated mononuclear cells per vessel circumference was assessed using analySIS Software 5.0 (Soft Imaging System, Muenster, Germany) by means of user‐driven skeletonization of the vascular circumference.

### 2.7 Bone marrow mononuclear cells isolation

To isolate bone marrow mononuclear cells, 4‐ to 6‐week‐old male BALB/c mice (BALB/cByJ; Charles River Laboratories, Freiburg, France) were sacrificed and their femurs and tibias collected in DMEM (Gibco) supplemented with 10% foetal bovine serum (FBS, Sigma‐Aldrich). Bone‐marrow was flushed‐off using PBS with 2% FBS and collected in a tube with 2 mmol/L ethylenediaminetetraacetic acid/PBS. The solution was then diluted with 10x volume RPMI1640 containing 0.02% collagenase B and 100 U/mL DNase and agitated gently at room temperature for 45 minutes. The cells were passed through 30 µm nylon mesh, centrifuged and resuspended in PBS. The cell suspension was gently and slowly added onto a tube containing the same volume of Histopaque‐1077 (Sigma Aldrich, Cat. 10771). After 30 minutes centrifugation, the white cellular ring floating over the Ficoll phase (mononuclear cells) was collected and transferred into a new tube, filled with 1X PBS and centrifuged to wash. At the end cells were re‐suspended in the appropriate medium.

### 2.8 Cell staining

The stock solution of CellTracker^TM^ Green 5‐Chloromethylfluorescein diacetate (10 mmol/L) was diluted to a final working concentration of 5 µmol/L in serum‐free medium. The working solution was warmed to 37°C. The mononuclear BMDC were centrifuged to pellet them and aspirate the supernatant. The cells were gently re‐suspended in pre‐warmed working solution, then incubated for 15‐45 minutes at 37°C and centrifuged. The probe solution was replaced with fresh, pre‐warmed medium and incubated for another 30 minutes at 37°C.

### 2.9 Adhesion assay

To perform adhesion assay, 10^5^ labelled mononuclear BMDC were add per well of cultured endothelial cells (on coverslips) for 15 minutes. The wells were washed with PBS, coverslips were fixed with 3.7% formaldehyde for 15 minutes at room temperature, stained with Hoechst dye for 10 minutes, rinsed with water and mounted on a slide for counting by microscopy.

### 2.10 Mononuclear cells isolation from CAV

Blood was washed out of the vessels of selected CAV samples. This was accomplished by injection of PBS. The treated tissue area was removed; making sure that for each embryo investigated the size of the tissue was the same. Tissues from at least 10 embryos for each test were then collected for each treatment and for controls. The tissue was cut into small pieces, digested in 500 µL 0.25% trypsin solution and 10 µL DNAse for 5 minutes at 37°C. The cell suspension was filtered through nylon gauze with a defined pore diameter of 30 µm, filled with 1X PBS and an equal volume of Histopaque‐1077 was added gently. After 30 minutes of centrifugation, the white cellular ring floating over the Ficoll phase (containing mononuclear cells) was collected, filled with 1X PBS and centrifuged for 10 minutes at 400 g to wash.

### 2.11 RNA isolation and Real‐time reverse transcription‐PCR

Total RNA was extracted from collected mononuclear cells from treated and control CAVs, by using High Pure RNA Cells Isolation Kit (Roche Diagnostics GmbH, Manheim, Germany). The RNA concentrations were determined spectrophotometrically. cDNA was synthesized by reverse transcription (1 hour at 37°C) using 500 ng of total RNA, 2.5 µmol/L of the oligo‐dT primers and 10 U of Reverse Transcriptase (Transcription High Fidelity cDNA Synthesis Kit, Roche Diagnostics GmbH). The following primer/probe kits (Chicken QuantiTest Primer Assay) were purchased from Qiagen: Chicken QuantiTest Primer Assay KDR2 (QT01140244), VEGFR1 (Flt1) (QT00595987), SDF‐1 (CXCL12) (QT01139026), CXCR4 (QT00590128), Tie‐2 (TEK) (QT00685629), Hes‐1 (QT00589015) and FGF2 (QT00599998). Quantitative PCR was performed using Real Time PCR (Applied BioSystems 7500 Fast). The fold change in expression levels of cDNA were determined from the ∆Ct values obtained as compared to ∆Ct values of control samples. In our experiment we used GAPDH (QT00588973) as housekeeping genes.

### 2.12 Immunofluorescence

Sections from liver samples were obtained at 5 μm, deparaffinized, and rehydrated. On dewaxed and rehydrated slides heat induced epitope retrieval in citrate buffer pH6 (DakoCytomation) was carried out for 5 minutes to unmask the epitopes. This was followed by blocking with 1% casein for 10 minutes. Incubation with the primary mouse antibody SDF‐1 and CXCR4 (Santa Cruz Biotechnology, Dallas, USA) overnight at 4°C (dilution 1:150) was followed by application of anti‐mouse IgG‐Cy3 (Sigma c2181) secondary antibody. Counterstaining was performed with Hoechst dye.

### 2.13 Statistical analysis

Probability associated with a Student's paired *t* test, with a two‐tailed distribution, was considered in a given p value for each comparison.

## 3 RESULTS

### 3.1 Effect of Notch inhibition on intussusceptive angiogenesis is mediated by SDF‐1/CXCR4 signalling

**Figure 1:**
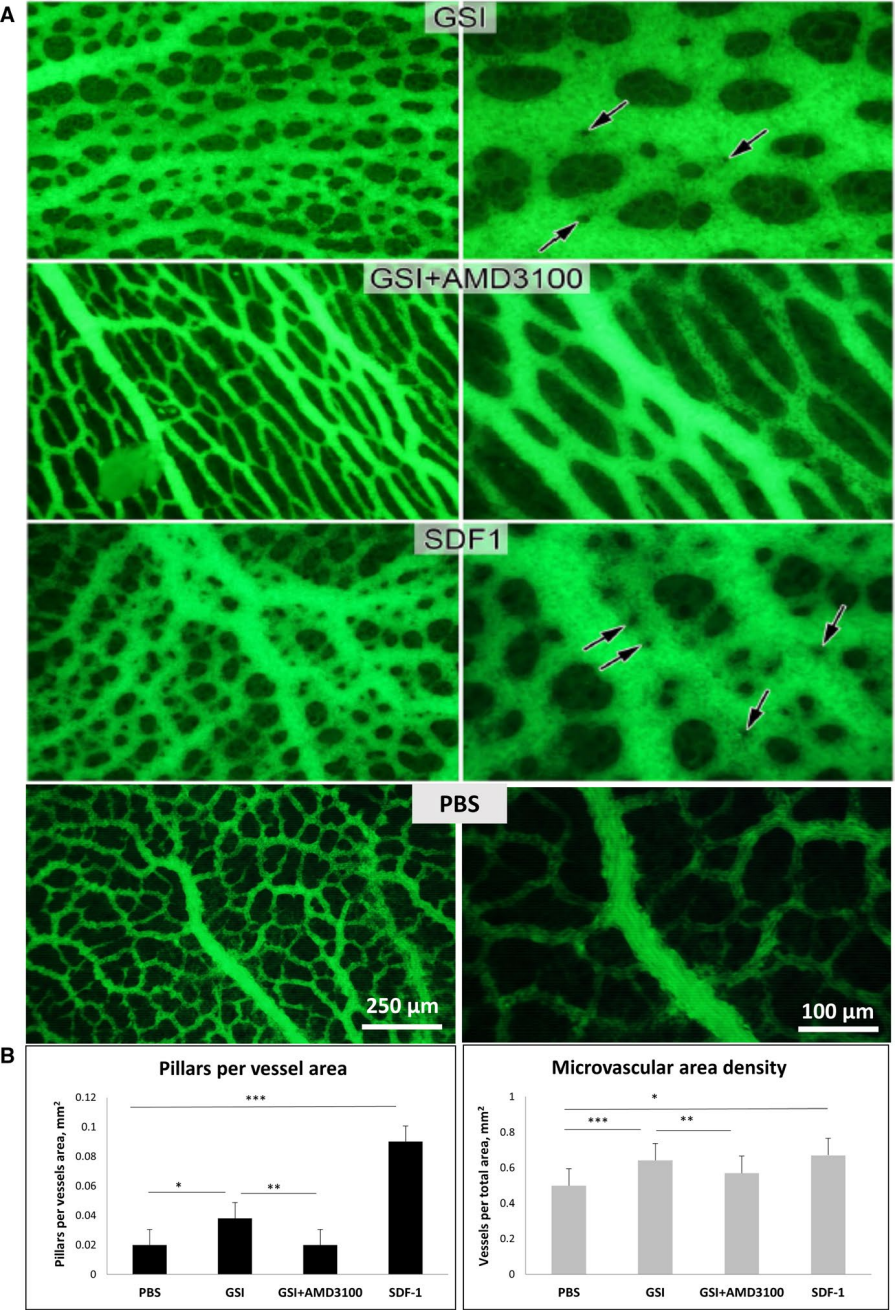
Effects of Notch inhibition on intussusceptive angiogenesis in the chick area vasculosa are mediated by SDF‐1/CXCR4 signalling. A, Fluorescein isothiocyanate microvasculature visualization after different targeted treatments. γ‐secretase inhibitor (GSI) application or treatment with recombinant SDF‐1 protein induced remarkably pillar formation (arrows), that is, intussusceptive angiogenesis. AMD3100 (CXCR4 antagonist) simultaneously applied with GSI demonstrated repressive effects on pillar formation. B, Bar graphs representing pillar density (*P < 10^-5^, **P < 10^-5^, ***P < 10^-6^) and vessel area density (*P < 0.01, **P < 0.01, ***P < 0.01) after different applications

We first analysed the formation of extra‐embryonic vasculature in CAV after simultaneous application of GSI (a potent Notch inhibitor) and AMD3100—an antagonist of CXCR4 receptor. Compared to the GSI application alone (Figure 1A), we detected two times less number of pillars along the vessels, thus indicating repressive effect on intussusceptive microvascular growth (Figure 1A). In contrast, treatment with recombinant SDF‐1 protein remarkably promoted intussusceptive angiogenesis in the same model, as is visible from the augmentation of pillar number by 4.5 times compared to control samples (Figure 1B). The microvascular density increased by 34% as well.

**Figure 2:**
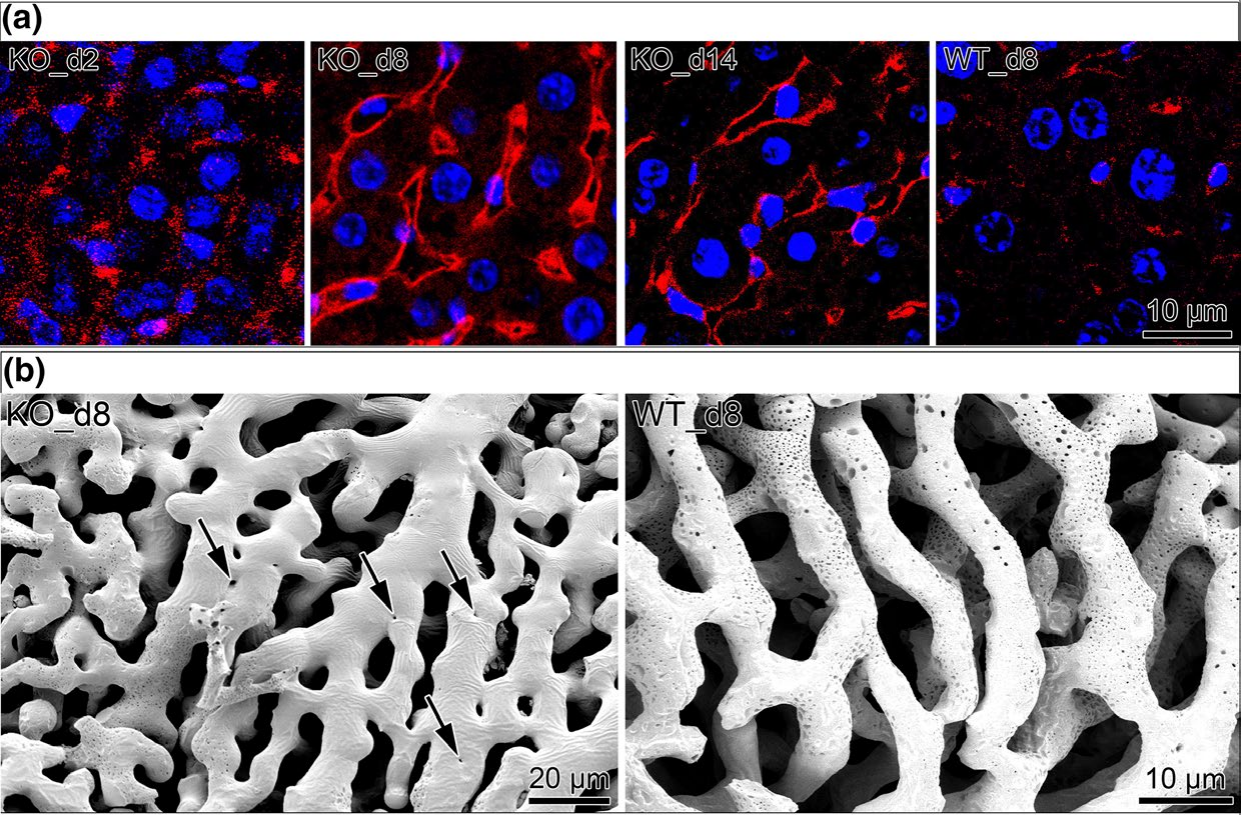
A, Significant elevation in SDF‐1 sinusoidal expression, assessed by immunohistochemistry, (red signal) in line with intussusceptive remodelling in Notch1 knockout mice. Nuclei stained in blue. B, Vascular casts revealed intensive vessel remodelling in Notch1 knockout mouse liver at the same time point. Pillars are indicated by arrows

If vascular‐derived SDF‐1 expression plays an important role in inducing intussusception, especially after Notch inhibition, we might expect that in Notch1 knockout (KO) mouse the sinusoid vessels would express high levels of this chemokine before and during intussusceptive remodelling. In accordance with this prediction, we detected by immunohistochemistry a mosaic distribution of SDF‐1 protein in sinusoid vessels of Notch1 KO mouse liver already at day 2 after Notch depletion (Figure 2). By day 8 its expression was already extensively spread on all sinusoids with a high intensity and remained stable for the next week (Figure 2). In comparison, WT mouse liver did not present substantial expression for SDF‐1 during this period (Figure 2). These results suggest that vessel‐derived SDF‐1 may be responsible for intussusceptive vascular remodelling in the liver of Notch1 KO mouse. Similarly, vascular casts revealed intensive intussusceptive vessel remodelling (intussusceptive pillars represented as a small holes in the vascular casts) in the liver of Notch1 KO mouse at the corresponding time points (Figure 2; Figure S1).

We performed immunofluorescent analysis for CXCR4 expression simultaneously with this one for SDF‐1 in Notch1 KO mouse and positivity for the receptor was evident in the mononuclear cells recruited to the SDF‐1‐positive sinusoidal vessels (Figure S2).

### 3.2 Attraction of mononuclear cells to the endothelium and their plausible role in vessel remodelling

**Figure 3:**
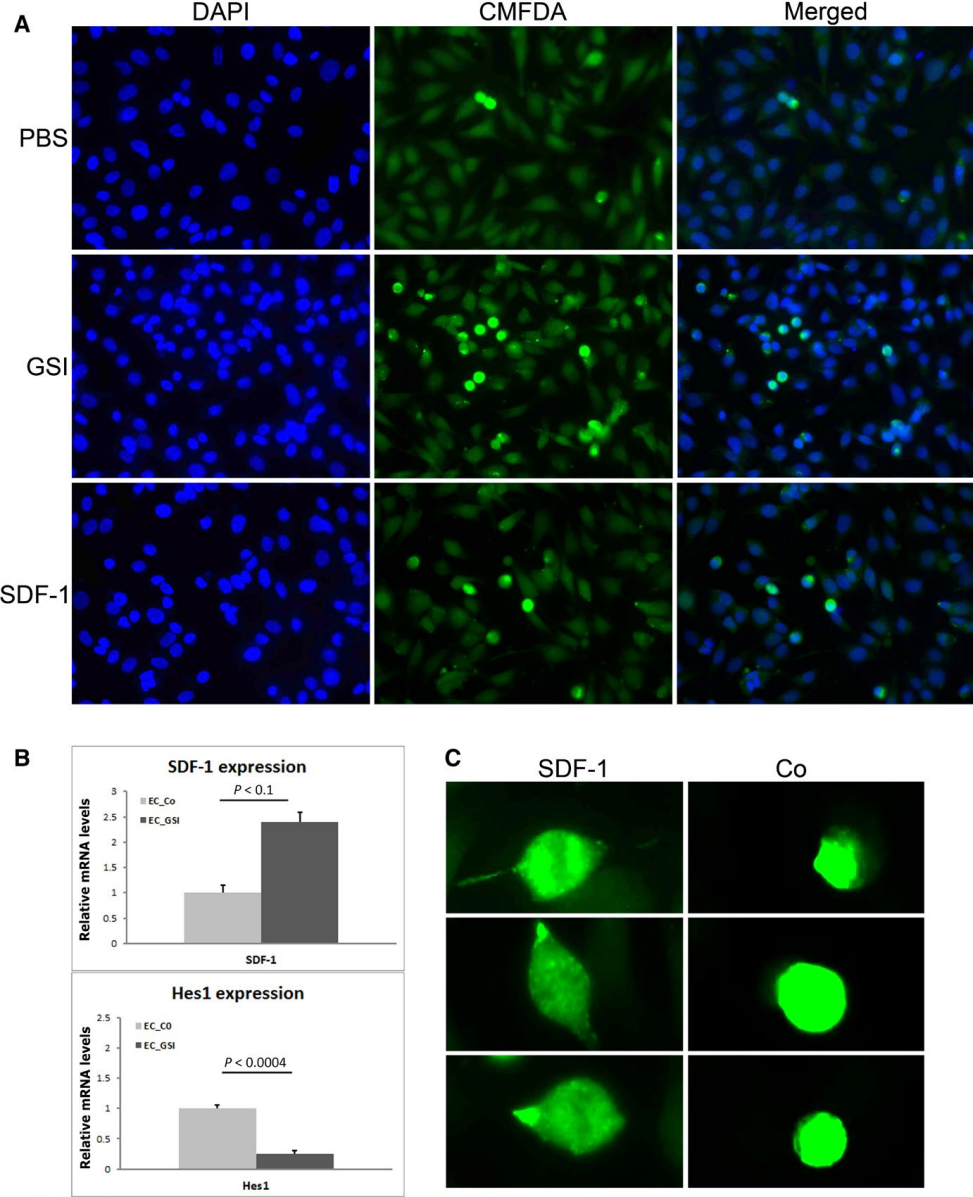
Adhesion assay. A, Mouse bone marrow cells, labeled with the green cell tracker 5‐Chloromethylfluorescein diacetate (CMFDA), adhere preferably to endothelial cells (blue 4′,6‐diamidino‐2‐phenylindole dihydrochloride (DAPI) staining) after γ‐secretase inhibitor (GSI) and SDF‐1 treatment. B, Increase in SDF‐1 expression after successful GSI blocking, assessed by expression of Hes1. C, Shape alterations of bone marrow‐derived mononuclear cells under SDF‐1 treatment

To examine one of the main functional effects of SDF‐1—attraction of mononuclear cells, we did in vitro experiment with co‐culture of bone marrow monocytes and endothelial cells. We were interested to see if the adhesion of BMD cells to the endothelium is comparable between the Notch inhibition subjects and SDF‐1 treatment ones. The effect of adhesion after Notch inhibition and SDF‐1 treatment was assessed by labelling mononuclear cells with Cell Tracker™ green and fluorescent microscopy. We detected comparable number of adherent mononuclear cells after GSI and SDF‐1 treatment on endothelial cells (Figure 3A; Figure S3). The inhibition of endothelial cells in culture (HUVEC) by GSI led to more than two times increase in SDF‐1 endothelial expression (Figure 3B). Successful GSI blocking was assessed by analysing the expression of Hes1 which is the most popular Notch target gene. These results suggest that the recruitment of mononuclear cells to the endothelium in GSI samples is due to SDF‐1/CXCR4 signalling. There were considerable shape alterations in mononuclear cells due to SDF‐1 application (Figure 3C).

**Figure 4:**
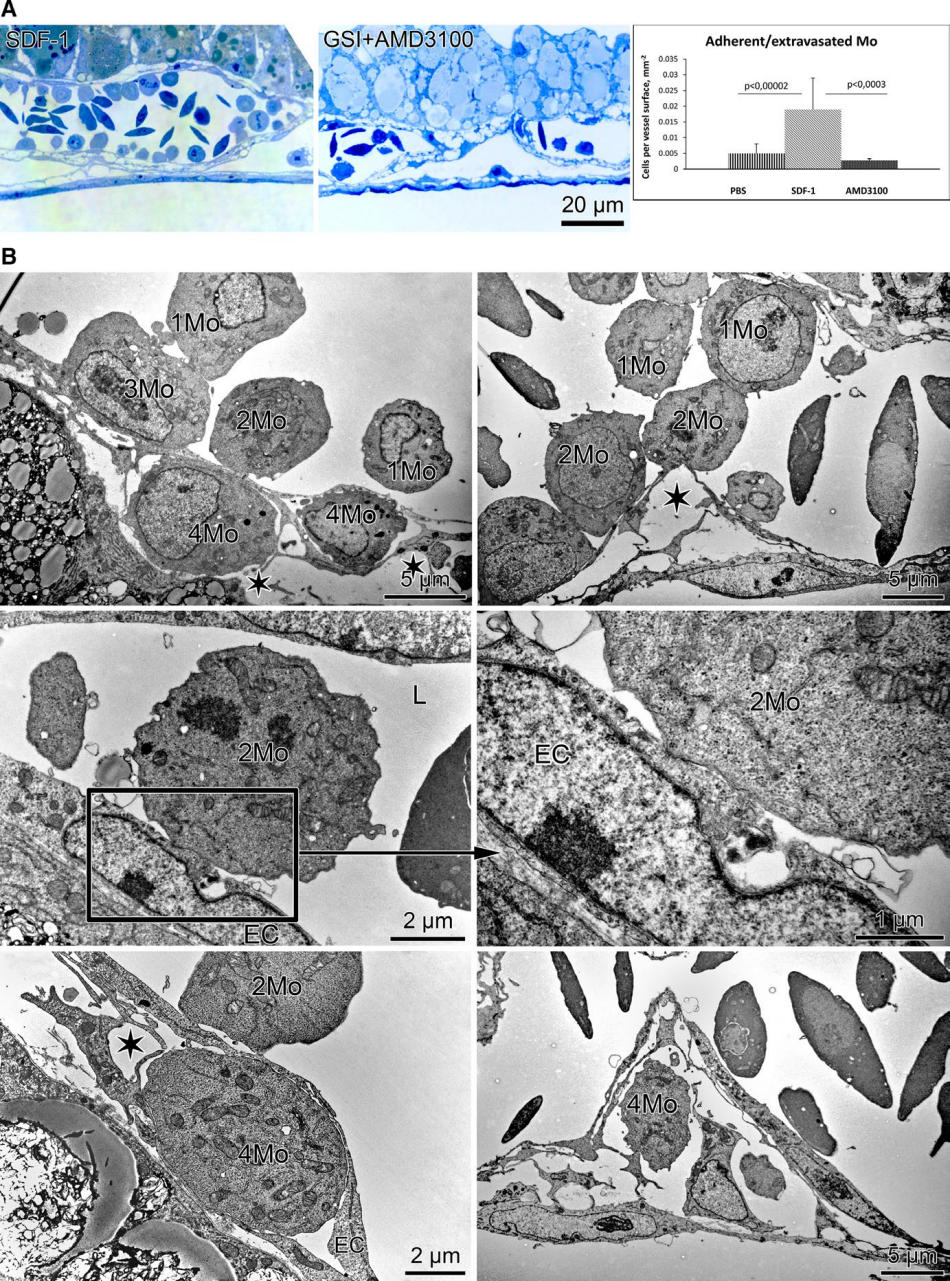
Area vasculosa adhesion/extravasation assay. A, Semithin section and quantification of adherent/extravasated cells per vessel area revealed mononuclear cells (Mo) attraction and recruitment after SDF‐1 application and inhibition of the process by AMD3100. B, Plausible roles of mononuclear cells in vascular recruitment and intussusceptive angiogenesis after SDF‐1 treatment. 1, circulating Mo; 2, Mo attachment; 3, Mo recruitment; 4, Mo extravasation; L, lumen; EC, endothelial cells; asterisks, EC‐pericytes detachment space enabling Mo extravasation

Further detailed structural analysis of the vessels was done on semithin sections. The number of adherent/extravasated mononuclear cells after SDF‐1 and its blockade by AMD3100 (following GSI treatment) in vivo was quantified (Figure 4A). A marked reduction of adherent/extravasated cells was observed after blockade of SDF‐1/CXCR4 signalling in GSI samples (Figure 4A).

A detailed ultrastructural analysis of the vessels after SDF‐1 treatment in order to investigate the intercellular interactions at a higher resolution was performed. Consistent with our hypothesis from a previous study for the extravasation of mononuclear cells and their participation in pillar formation after Notch inhibition, we detected different steps in mononuclear cell behaviour after SDF‐1 application: recruitment, attachment and extravasation (Figure 4B). Close contacts were observed between mononuclear cells in the lumen and endothelial cells from the opposite sites (Figure 4B); the extravasated mononuclear cells were integrated between the basement membrane and the pedicels of pericytes (Figure 4B); the attached/extravasated mononuclear cells make retraction/invagination of the basement membrane into the vessel lumen (Figure 4B).

### 3.3 Angiogenic expression pattern of extravasated mononuclear cells

**Figure 5:**
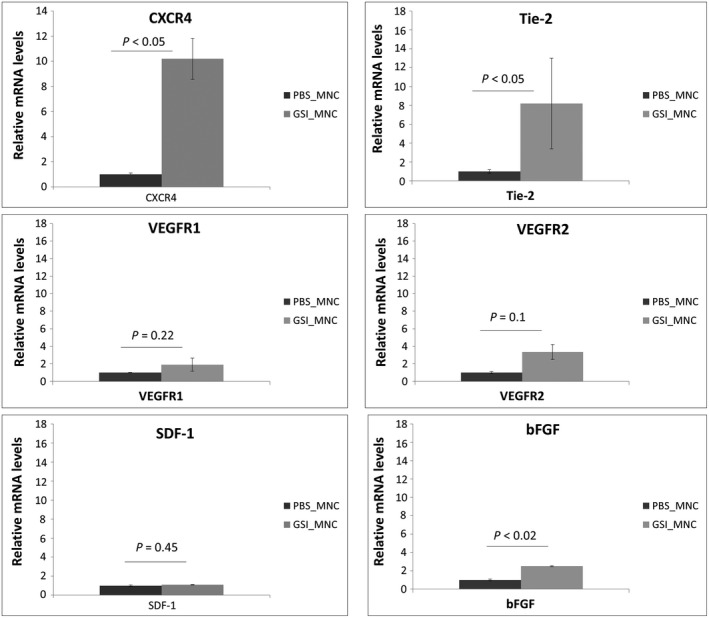
Total fold change in cDNA expression level profiles for receptors CXCR4, Tie‐2, VEGFR1 and VEGFR2, and for growth factors SDF‐1 and basic fibroblast growth factor (bFGF) in the exstravasated mononuclear cells (MNC) after PBS and γ‐secretase inhibitor (GSI) application in the area vasculosa

Since the morphological analysis does not give us information about the molecular phenotype of the mononuclear cells, we specifically isolated these cells after GSI application from the in vivo model (CAV) and tried to characterize their expression pattern for some specific angiogenic markers, especially surface receptors (Figure 5). Quantitative measurement revealed significantly higher expression for CXCR4 membrane receptor in mononuclear cells from treated samples compared to controls, as well as to the same extent higher expression levels for Tie‐2 receptor, suggesting that these cells are CXCR4^+ ^and Tie2^+^. Expression of bFGF was increased by 2.5 times in mononuclear cells after treatment and no changes were detected for SDF‐1 expression. The expression levels for VEGFA receptors 1 and 2 did not show significant differences. These data, together with the results for vessel SDF‐1 expression, assumed that the recruitment of mononuclear cells during the process of intussusception is exerted by the interplay between CXCR4 receptor on the mononuclear cells' surface and SDF‐1 ligand expressed by the endothelium. The role of these cells is rather physical for pillar formation by supporting the intraluminal invagination of the endothelium. Their positivity for Tie‐2 opened the question for the involvement of angiopoietin‐2 signalling in this process.

### 3.4 Positive feedback relation between SDF‐1 expression and shear stress

**Figure 6:**
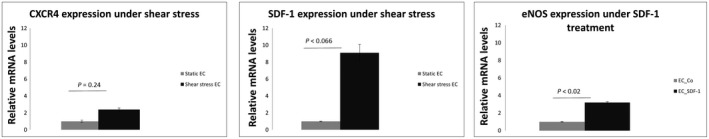
Positive feedback relation between SDF‐1 and shear stress assessed by increase in SDF‐1 expression under shear stress (A) and increase in nitric oxide synthase expression under SDF‐1 treatment (B)

As intussusceptive angiogenesis can be initiated only in the presence of blood flow, an important aspect is the molecular link between shear stress and its effects on the regulation of angiogenesis. As the focus of our study here, we wanted to investigate the expression of SDF‐1 under an exposure of specifically determined shear stress. Human umbilical vein endothelial cells were seeded into 75 cm^2^ plates and grown to confluence in DMEM supplemented with 10% FBS, 1% antibiotic. After three passages the cells were seeded on 1 u‐Slide (y‐shaped ibiTreat). After 24 hours the shear stress of 20 dyne/cm^2^ was applied for 3 hours, the slides without shear stress were kept static for the same time to be used as control. The cells afterwards were collected in RNA Latter for RNA extraction. We detected high increase (nine times) in the expression levels of SDF‐1 under the applied shear stress (Figure 6A). Inversely, we tested if application of SDF‐1 on endothelial cells increases the expression of eNOS—a marker of increased shear stress. We found more than three times increase in eNOS expression levels (Figure 6B). These results suggest a positive feedback loop between shear stress and SDF‐1, each of them inducing the other. If this is the case, it could be the possible explanation for the rapid expansion of intussusception during the growth and remodelling of the vasculature. Further investigations are needed to clarify this observation.

## 4 DISCUSSION

Several studies in the recent past allowed distinguishing of subpopulations of mononuclear cells existing in the adult bone marrow and circulating in peripheral blood that support angiogenesis without being incorporated permanently into the newly formed vessels, the so‐called circulating angiogenic cells (CAC).^34^ Here, we showed that mononuclear cells of bone marrow origin are recruited to vessels in response to SDF‐1 endothelial expression. Our previous studies demonstrated that extravasating mononuclear cells stabilize pillars, the hallmarks of intussusception, by formation of uropod‐like protrusions and collagen production.^16^ In the current study we have found that they express bFGF, supporting the previous observation. In addition, they were highly positive for the expression of CXCR4 and Tie‐2 receptors. Pharmacological inhibition of SDF‐1/CXCR4 signalling by small CXCR4 antagonist AMD3100 perturbs intussusceptive vascular growth (two times less pillar number) and abolishes mononuclear cell recruitment in GSI treated samples of CAV. In contrast, treatment of CAV by recombinant SDF‐1 protein increased microvascular density by 34% through augmentation of pillar number by 4.5 times compared to control samples. The number of extravasating mononuclear cells was four times higher after SDF‐1 application and two times less after blocking this signalling pathway. Similarly, Butler et  al showed that administration of recombinant SDF‐1 protein within the vitreous chamber of murine model of proliferative diabetic retinopathy^35^ promoted neovascularization and SDF‐1 is both necessary and enough for inducing proliferative retinopathy. This finding was supported clinically by detection of high vitreal SDF‐1 concentration in patients with diabetic retinopathy. The murine model used GFP + HSC‐derived progenitor cells and proved their migration and incorporation into the sites of ischaemic injury after SDF‐1 application. The latter authors, however did not highlight the role of the cells in the development of functional blood vessels. SDF‐1 expression was also up‐regulated in a hypoxia‐induced pulmonary hypertension mouse model.^36^ Inhibition of SDF‐1/CXCR4 axis decreases progenitor cells in the lungs, at the same time prevents and reverses pulmonary vascular remodelling. GFP+ cells (GFP‐BMDC?) were visualized in the smooth muscle and adventitial layers of the hypoxic pulmonary arteries. In our previous study, after Notch inhibition we detected mononuclear cells extravasating and participating in the formation of pillars during intussusceptive angiogenesis, which is a main mechanism of vascular adaptation. Here we confirm that extravasation and recruitment of BMDC is due to increased expression of SDF‐1, and occurs concomitantly with intussusceptive angiogenesis both in the CAV and in the mouse model. Ultrastructural investigation under TEM combined with molecular analyses suggest, that the BMDC are CXCR4‐positive and are as such attracted to the SDF‐1‐positive endothelium and physically support intraluminal endothelial invagination. In the CAV model with simple tumour‐like vessels we observed how the BMDC are integrated between endothelial cells and the pericytes. Further looking at their morphology, we noticed formation of uropod‐like structures during their adherence to the endothelium. The in vitro experiments indicated the same extent of BMDC adhesion after GSI and SDF‐1 treatment and formation of uropods after SDF‐1 application, assuming the adhesion and uropod formation were mainly due to SDF‐1. Renewed interest in cell shape has been prompted by a recent abundance of evidence indicating that cell polarity is essential for the biology of motile cells. Uropod is defined as a protrusion of motile cells, important for intercellular adhesion, cell migration, apoptosis and vesicle trafficking. Recently the mechanism of uropod formation was described, as the triggering event is cell polarization by chemotaxis molecules.^37^ SDF‐1 regulates adhesion, motility and cell shape in tumour progression.^38^ They described morphological changes from round to polygonal shape, including the formation of neurite‐like projections, increased membrane ruffling, and more frequent filopodia and uropod formation in response to SDF‐1. We suggest that SDF‐1 influences protrusion formation in mononuclear cells, facilitating their participation in pillar development during intussusceptive angiogenesis. This is the first study reporting connection between SDF‐1 endothelial expression and intussusceptive mode of angiogenesis. A comprehensive study for SDF‐1 expression in vessels was done by Salvucci et  al.^39^ They detected the presence of SDF‐1 in endothelial cells of capillaries in bone marrow and skin, as well as in the endothelium lining umbilical veins, the chorionic villi, and the high endothelial venules in lymph nodes. Although SDF‐1 is not expressed in the endothelia of capillaries from many organs in normal conditions (such as kidneys, brain, skeletal muscles, lung and liver), it is encountered in the capillaries from the same organs in pathological conditions, such as glioblastoma multiforme, infarcted brain tissue, Burkitt lymphoma tissue and lobular capillary hemangioma. This suggests that SDF‐1 expression could be induced in endothelial cells during new vessel formation. We demonstrated SDF‐1 positivity in the endothelium of liver sinusoidal vessels of Notch1 KO mice, representing regenerative hyperplasia by means of intussusception, but SDF‐1 was not detected in WT mice with normal liver structure. The intraluminal endothelial protrusions were also positive for SDF‐1 in the case of capillary hemangioma, described in the above‐mentioned study.^39^

SDF‐1, when expressed in the bone marrow and various tissues, is able to regulate trafficking, localization and function of immature and mature leukocytes, including monocytes, neutrophils, dendritic cells and T lymphocytes.^40^ All these immune cells play important roles in tumour angiogenesis and vascularization. It is well known that blocking of SDF‐1/CXCR4 axis results in prevention or delay of tumour recurrence after irradiation by inhibiting the recruitment of CD11b+ monocytes/macrophages that participate in tumour revascularization.^41^ SDF‐1/CXCR4 signalling has pivotal role in mast cell (MC) recruitment in tumour tissue^42^ and MC produce pro‐angiogenic chemokines in response to SDF‐1,^43^ thus exerting important angiogenic activity. Mononuclear cells adherence to endothelium was four times more after treatment with recombinant SDF‐1 protein. For therapeutic purposes, improvement of monocyte recruitment to the injured site might be more suitable than injection of monocytic cells in circulation, which results in only slight increase in the number of circulating monocytes. We demonstrated that blocking SDF‐1/CXCR4 signalling clearly abrogated the recruitment of mononuclear cells to the vessels, at the same time reducing pillar formation respectively intussusceptive angiogenesis.

Despite the important role of intussusception in vessel formation and remodelling, most of the existing studies are focused on the better known mechanism of sprouting angiogenesis. Thus, the mechanism of intussusceptive angiogenesis has not been adequately covered contemporary by investigators in the field of angiogenesis research. Intussusception is an alternative to the sprouting mode of angiogenesis.^44^ The advantage of this mechanism of vascular growth is that blood vessels are generated more rapidly and the capillaries thereby formed are less leaky.^45^ Regarding molecular regulation, very little is known of the molecular factors with potential significance in intussusceptive angiogenesis. Application of the essential angiogenic factors VEGF and bFGF in the arteriovenous loop model demonstrated advanced neovascularisation in the phase of remodelling by a higher incidence of intussusception, compared to controls.^46^ It was found that neovascularization induced by VEGF plus bFGF is mediated by SDF‐1/CXCR4 signalling and that SDF‐1 neutralization reduced growth factor‐triggered neovascularization by approximately 84%‐86%.^39^ In addition, our experiments on HUVEC demonstrated positive feedback between shear stress and SDF‐1. Recently, it was proposed that Notch1 is atheroprotective and acts as a mechanosensor in adult arteries, where it integrates responses to laminar shear stress,^47^ as well as that chemokine receptor CXCR4 pathway is the key regulator of Notch‐dependent vessel growth.^48^

In conclusion, our study is the first to implicate SDF‐1/CXCR4 signalling in the mechanism of intussusceptive angiogenesis. Plausibly, BMDC play an important physical and stabilizing role in the formation of pillars during vascular augmentation.

## CONFLICT OF INTEREST

The authors confirm that there are no conflicts of interest.
