# Supplementary figures and images for: SDF‐1/CXCR4 signalling is involved in blood vessel growth and remodelling by intussusception
Source: J Cell Mol Med. 2019 Apr 4;23(6):3916–26. doi: 10.1111/jcmm.14269 (PMC6533523; doi:10.1111/jcmm.14269)

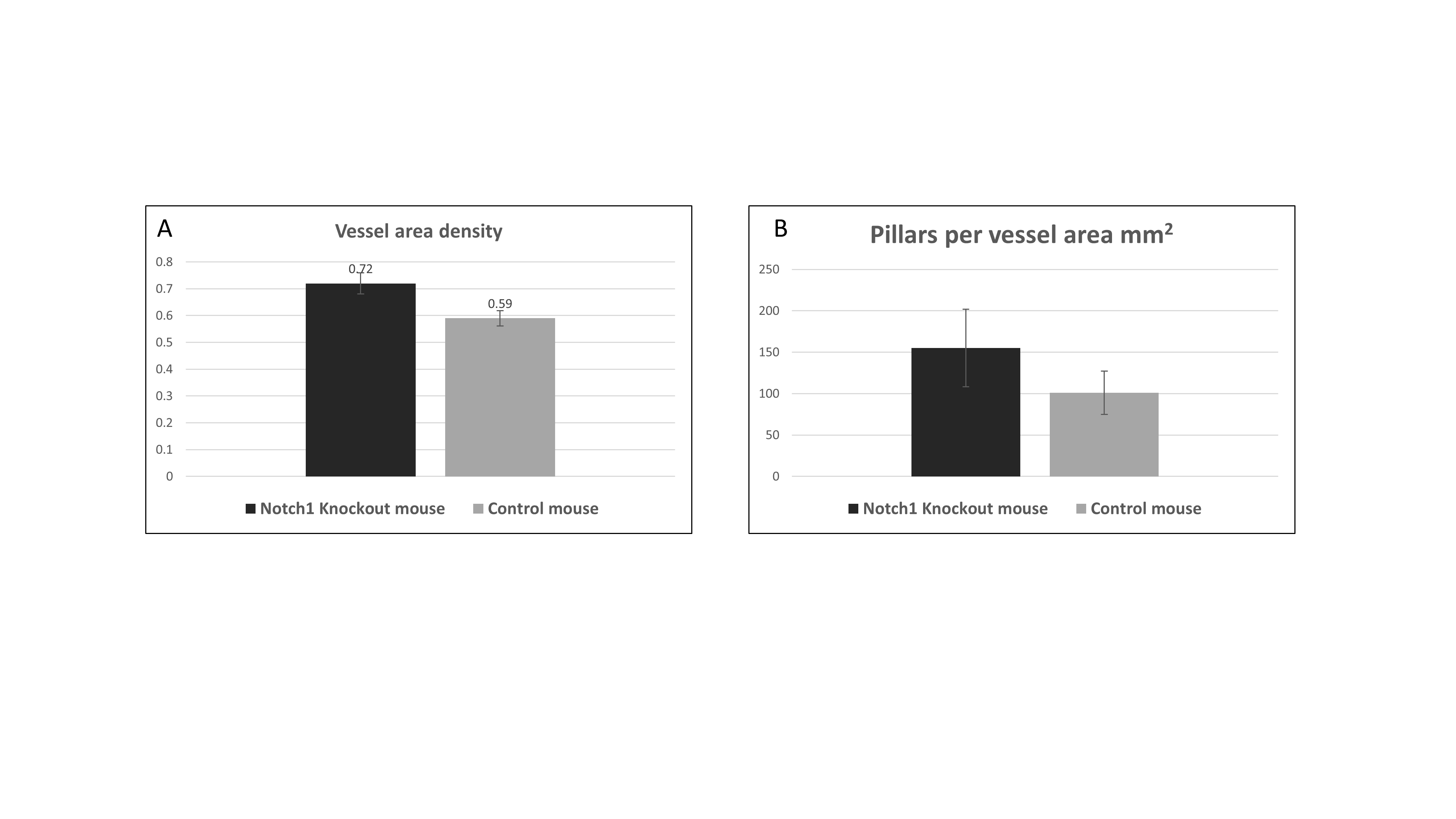

Supplement: Supplementary file 1 [file JCMM-23-3916-s001.tif]

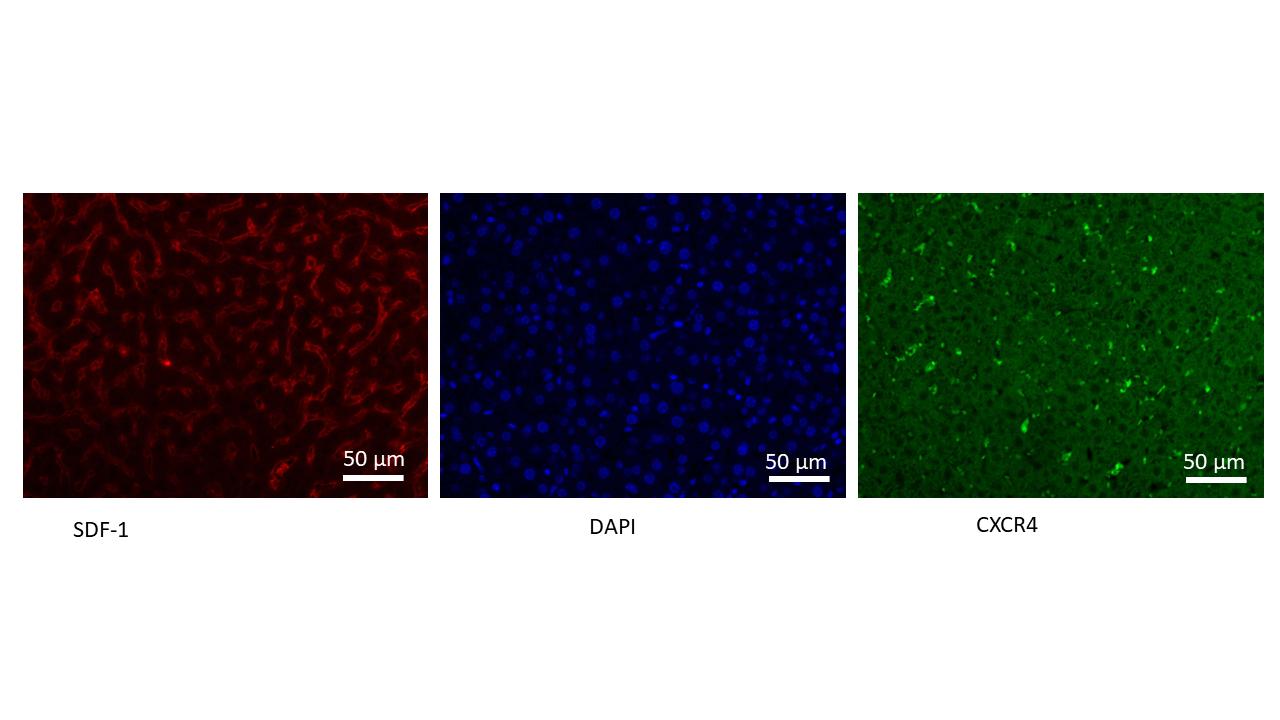

Supplement: Supplementary file 2 [file JCMM-23-3916-s002.tif]

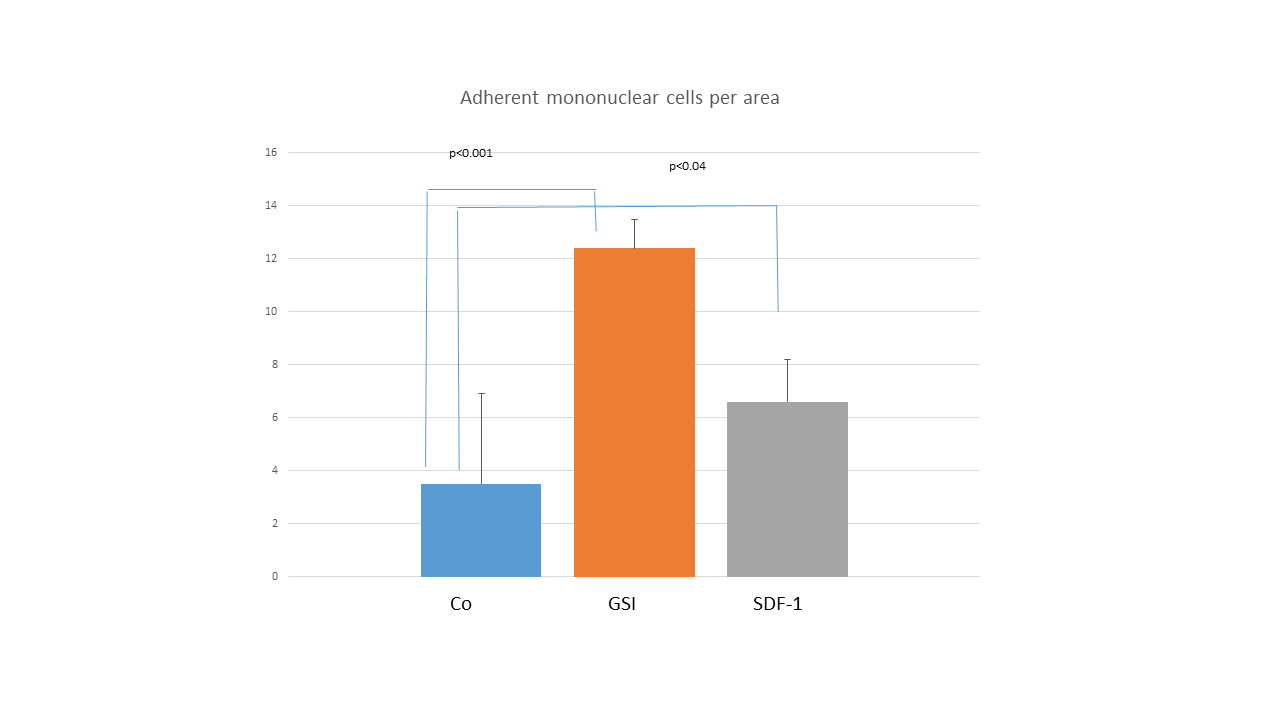

Supplement: Supplementary file 3 [file JCMM-23-3916-s003.tif]
