# Supplementary material for: SDF‐1/CXCR4 signalling is involved in blood vessel growth and remodelling by intussusception
Source: J Cell Mol Med. 2019 Apr 4;23(6):3916–26. doi: 10.1111/jcmm.14269 (PMC6533523; doi:10.1111/jcmm.14269)
Supplement: Supplementary file 4 [file JCMM-23-3916-s004.docx]

**Figure S1**. Estimation of intussusceptive microvascular growth in Notch1 knockout (KO) mouse. A. Microvascular area density increased in the liver of KO mouse compared to control (p<0.05); B. Pillar density increased significantly in the liver KO mouse compared to control (p<0.02).

**Figure S2**. Immunofluorescence revealed SDF-1 sinusoidal expression in the liver of Notch1 knockout mouse (red signal) and CXCR4 expression from the adjacent cells (green signal). Nuclei stained in blue.

**Figure S3**. The number of adherent mononuclear cells to the endothelial cells (ECs) was significantly higher in GSI treated ECs (p<0.001) and in SDF-1 treated ECs (p<0.04) compared to non-treated ECs.
